# Cytotoxicity of Nine Medicinal Plants from San Basilio de Palenque (Colombia) on HepG2 Cells

**DOI:** 10.3390/plants12142686

**Published:** 2023-07-19

**Authors:** Karina Caballero-Gallardo, Neda Alvarez-Ortega, Jesus Olivero-Verbel

**Affiliations:** 1 Environmental and Computational Chemistry Group, School of Pharmaceutical Sciences, Zaragocilla Campus, University of Cartagena, Cartagena 130014, Colombia; nalvarezo@unicartagena.edu.co (N.A.-O.); joliverov@unicartagena.edu.co (J.O.-V.); 2 Functional Toxicology Group, School of Pharmaceutical Sciences, Zaragocilla Campus, University of Cartagena, Cartagena 130014, Colombia

**Keywords:** extracts, traditional medicine, biodiversity, inflammation

## Abstract

The utilization of plants with medicinal properties is deeply rooted in the traditional knowledge of diverse human populations. This study aims to investigate the cytotoxicity of nine plants commonly used by communities in San Basilio de Palenque, Bolivar (Colombia), for managing inflammation-related illnesses. Hydroethanolic extracts from various plant parts such as roots, stems, barks, or leaves were prepared through a process involving drying, powdering, and maceration in an ethanol–water (7:3) solution. The extracts were subsequently freeze-dried and dissolved in DMSO for the bioassays. Cytotoxicity against the human hepatoma HepG2 cell line was assessed using the MTT assay, with extract concentrations ranging from 0 to 500 µg/mL and treatment durations of 24 and 48 h. The total phenolic content of the nine extracts varied from 96.7 to 167.6 mg GAE/g DT. Among them, eight hydroethanolic extracts from *Jatropha gossypiifolia* L., *Piper peltatum* L., *Malachra alceifolia*, *Verbesina turbacensis*, *Ricinus communis*, *Desmodium incanum*, and *Dolichandra unguis-cati* showed low toxicity (IC_50_ > 500 µg/mL, 24 h) against HepG2 cells. On the other hand, the extracts of *Aristolochia odoratissima* L. (IC_50_ = 95.7 µg/mL) and *Picramnia latifolia* (IC_50_ = 128.9 µg/mL) demonstrated the highest cytotoxicity against the HepG2 cell line, displaying a modest selectivity index when compared to the HEKn cell line after 48 h of treatment. These findings suggest that medicinal plants from San Basilio de Palenque, particularly *Picramnia latifolia* and *Aristolochia odoratissima*, have potential activity against cancer cells, highlighting their potential for further research and development.

## 1. Introduction

Medicinal plants serve as valuable sources of new drugs globally [1]. In Europe, over 1300 medicinal plant species are utilized, with approximately 90% derived from wild resources [2]. In the United States, a significant number of major prescription drugs are based on natural sources. The World Health Organization (WHO) reports that around 80% of the population in developing countries relies on traditional medicine for their healthcare needs [3]. The utilization of medicinal plants is rapidly increasing worldwide. However, due to overexploitation, habitat destruction, and the growing human population’s consumption of plants, approximately 15,000 species are currently threatened with extinction, and nearly 20% of their wild resources have already been depleted [4,5]. The American continent, particularly South America, is recognized for its vast cultural and biological wealth, hosting one of the largest areas of biological diversity globally. Approximately 40% of the continent’s biodiversity is concentrated in South America, particularly in its tropical forested regions [6].

The tropical forests of Colombia boast remarkable biodiversity of flora and fauna, thanks to their abundant rainfall, which is the highest among all the regions in the Americas. This climatic condition, characterized by a bimodal regime, directly supports diverse ecosystems and promotes biodiversity [7]. Unfortunately, these once-pristine ecosystems have faced increasing devastation over the past five decades due to human activities, particularly poor agricultural practices such as extensive livestock farming and monocultures. These practices potentially pose threats to native wild species that remain understudied [8]. Furthermore, despite their ecological importance, tropical forest ecosystems in Colombia lack comprehensive biological characterization, including detailed assessments of the diversity and distribution of the flora within these ecosystems. Limited research efforts have been devoted to inventorying the local flora, especially those with medicinal properties, and their influence on the ethnobotanical practices of surrounding communities [8].

The Montes de Maria region, located between the Bolívar and Sucre departments in the Colombian Caribbean subregion, harbors a tropical forest known for its remarkable biological diversity. However, the understanding of medicinal flora within this vital biological corridor is severely limited, let alone the knowledge of its local medicinal uses by ethnic communities who possess ancestral wisdom. The unique conditions of the region, compounded by the threats of climate change, pose significant risks to this ancestral knowledge. As the majority of the native flora grows in the wild, conserving the ethnobotanical biodiversity becomes even more challenging. Furthermore, a scarcity of scientifically grounded information hinders the characterization of the bioactive components present in this wild flora [8].

Sociocultural factors, including a deficit in traditional oral knowledge concerning the use of local flora with medicinal properties, along with forced displacement resulting from territorial conflicts, have significantly hindered access to and preservation of this invaluable knowledge. These combined challenges are contributing to the erosion of ancestral wisdom and the loss of cultural and intangible heritage, particularly among the primarily black communities and Afro-descendants who have settled in the region [9].

In the Afro-Colombian context, there exists a profound sociocultural connection between the utilization of local vegetation for medicinal purposes and the holistic understanding of the human organism within a cultural and spiritual system. This relationship between life and death, deeply embedded in the Colombian Caribbean region, has transcended its local significance to become a national phenomenon. As a testament to its cultural importance, San Basilio de Palenque has been recognized and designated by UNESCO as a Masterpiece of the Oral and Intangible Heritage of Humanity [10].

The international recognition bestowed upon San Basilio de Palenque is attributed, in part, to its rich culture and local heritage. Central to this heritage is the knowledge of ancestral medicine, which serves as a fundamental cultural and religious component with deep African roots. It represents an indigenous blend of Spanish and native traditions, creating a unique intangible heritage [10]. Despite its significance, this territory remains understudied in terms of understanding its ethnobotanical biological diversity for medicinal purposes, with only a few reports available [10].

Conversely, in recent decades, the preservation of ancestral medicine has been jeopardized by the scarcity of scientific understanding surrounding these practices, resulting in a shift towards conventional medicine. Moreover, processes of forced migration have led to the separation of ancestral knowledge from neighboring regions, resulting in fragmented knowledge within subregions. Unfortunately, these social circumstances have hindered the communities’ ability to reclaim their ancestral territories. The objective of this study was to assess the total phenolic, flavonoid, and tannin contents, antioxidant activity, and cytotoxicity of hydroethanolic extracts derived from nine plant species: *Aristolochia odoratissima* L., *Jatropha gossypiifolia* L., *Piper peltatum* L., *Malachra alceifolia* Jacq., *Picramnia latifolia* Tul., *Verbesina turbacensis* Kunth, *Ricinus communis* L., *Desmodium incanum* (Sw.) DC., and *Dolichandra unguis-cati* (L.) L.G. Lohmann. Additionally, the study aimed to evaluate the cellular-protective potential of hydroethanolic extracts from *A. odoratissima* L. and *P. latifolia* Tul. stems against the cytotoxicity induced by zearalenone (α-ZEL) and APAP on HepG2 cells.

## 2. Results

### 2.1. Total Phenolic, Flavonoid, Tannin Contents, and DPPH Free Radical Scavenging Activity

The quantification of total phenolic, flavonoid, and tannin contents, as well as DPPH free radical scavenging activity, is presented in Table 1. The phenolic contents of the hydroethanolic extracts ranged from 96.7 to 169.4 mg GAE/g DT, with the highest concentration observed in *A. odoratisimma* (167.6 mg GAE/g DT). The total flavonoid content varied from 6.9 to 95.1 mg RE/g DT, while *P. latifolia* exhibited the highest levels of condensed tannins (73.5 mg CE/g DT). Among the extracts, *D. incanum* (Sw.) displayed the highest DPPH free radical scavenging activity, with an IC_50_ value of 0.04 mg/mL.

**Figure 1:**
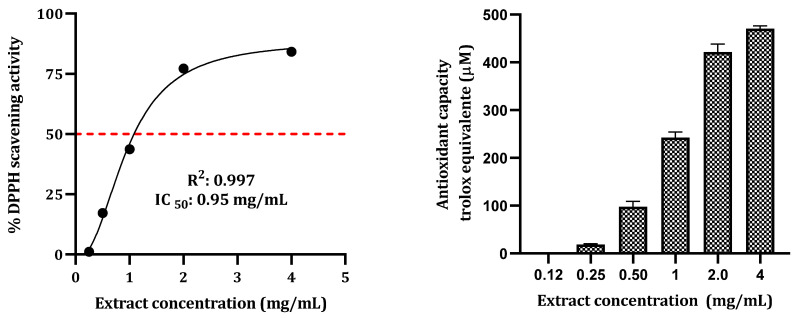
The DPPH radical scavenging capacity of the *P. latifolia* extract is presented in the (**left panel**), while the antioxidant capacity is measured in Trolox equivalents (µM) and displayed in the (**right panel**).

The DPPH free radical scavenging activity of *P. latifolia* is depicted in Figure 1. At a concentration of 4 mg/mL, the extract demonstrated a scavenging activity of 84.1%. The IC_50_ value for *P. latifolia* extract was determined to be 0.95 mg/mL. The antioxidant assay yielded values ranging from 20 to 476.6 µM Trolox.

### 2.2. Screening of Cytotoxic Activity of Hydroethanolic Extracts

**Figure 2:**
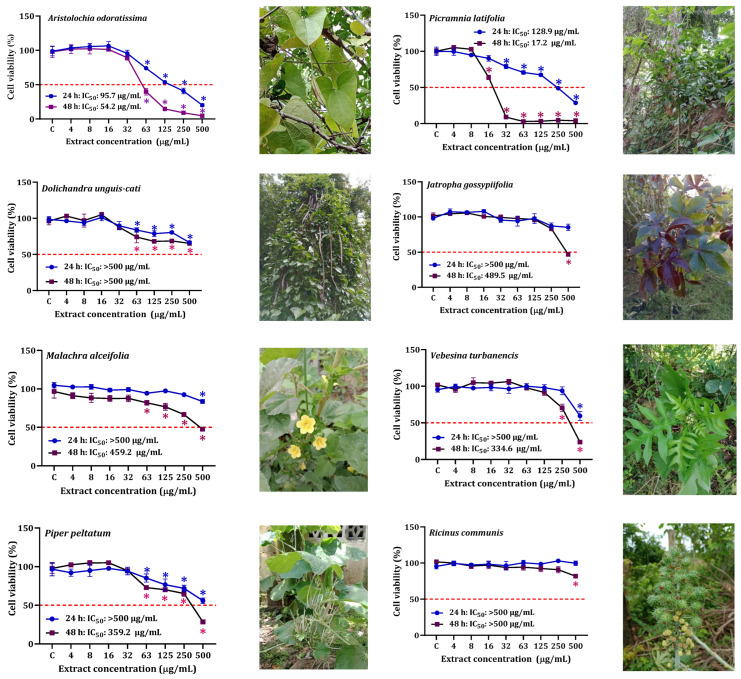

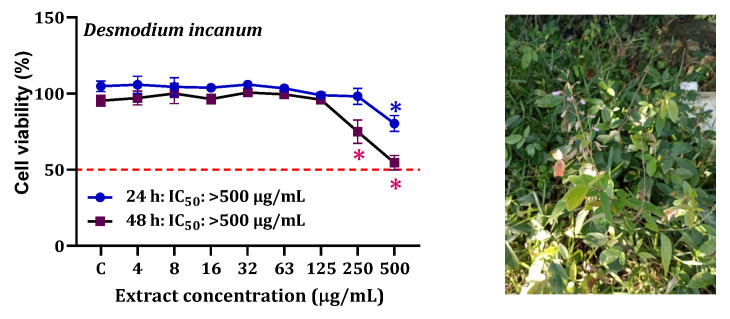
Cytotoxicity of nine hydroethanolic extracts on HepG2 cells after 24 and 48 h of exposure. * Significant difference (*p* < 0.05) when compared to the control group (C).

The cytotoxicity of the examined plant extracts on HepG2 cells is depicted in Figure 2. HepG2 cells exposed to the *P. latifolia* extract at concentrations ranging from 32 to 500 µg/mL showed a significant concentration-dependent reduction in cell viability, particularly after 48 h (IC_50_ = 17.2 µg/mL). In contrast, the other extracts exhibited lower cytotoxicity at both exposure times, with IC_50_ values exceeding 500 µg/mL.

**Figure 3:**
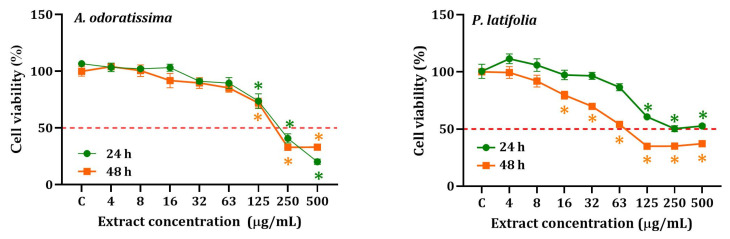
Cytotoxicity of hydroethanolic extracts of *A. odoratissima* and *P. latifolia* on HEKn cells after 24 and 48 h of exposure. * Significant difference (*p* < 0.05) when compared to the control group (C).

The IC_50_ values after 48 h of exposure were found to be low for the extracts of *P. latifolia* (IC_50_: 17.2 µg/mL) and *A. odoratissima* (IC_50_: 54.2 µg/mL). The results on the cytotoxicity of noncancerous cells (HEKn) exposed to *A. odoratissima* and *P. latifolia* extract and the selectivity index (SI) are shown in Figure 3 and Table 2. These extracts demonstrated lower cytotoxicity towards HEKn cells compared to HepG2 cells, with IC_50_ values of 38.9 µg/mL and 54.2 µg/mL for *P. latifolia* and *A. odoratissima*, respectively, after 48 h of exposure. Notably, the *P. latifolia* extract exhibited a more pronounced cytotoxic effect on cancer cells compared to non-cancer cells (SI: 2.3, 48 h). Furthermore, both extracts displayed moderate selectivity, as depicted in Figure 3.

### 2.3. Cytoprotective Effects of P. latifolia against α-ZEL and APAP

**Figure 4:**
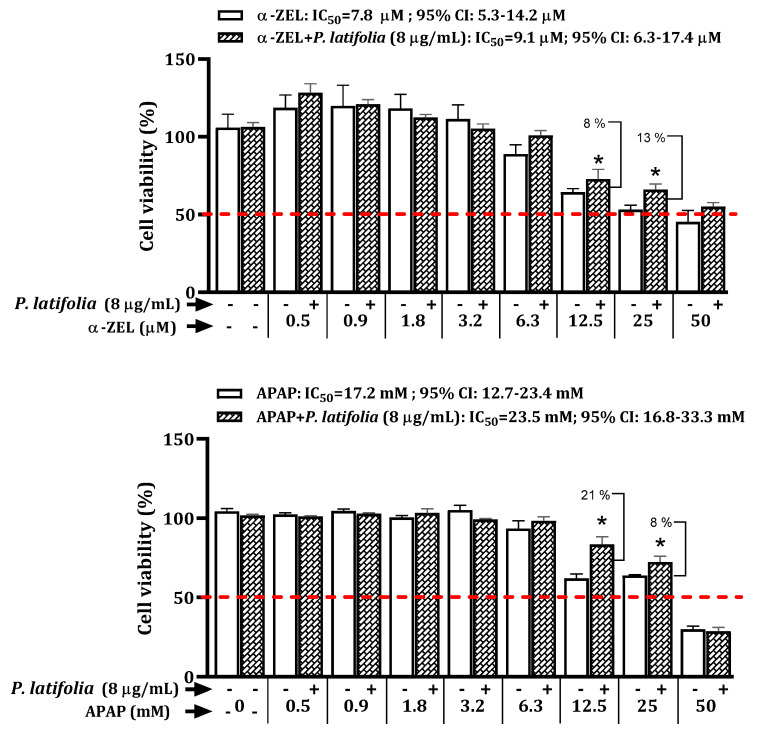
Cytoprotective effects of *P. latifolia* extract on HepG2 cells exposed to α-ZEL and APAP after treatment for 24 h. * Significant difference (*p* < 0.05) compared to the group treated with α-ZEL or APAP.

The protective effect of *P. latifolia* on α-ZEL and APAP after 24 h of exposure is depicted in Figure 4. The results demonstrated that simultaneous treatment of HepG2 cells with α-ZEL (12.5 and 25 μM) or APAP (12.5 and 25 mM) along with the extract (8 μg/mL), significantly (*p* < 0.05) preserved cell viability, with cytoprotection ranging from 8% to 13% and 21% to 8%, respectively. Additionally, it was observed that the IC_50_ remained similar under these conditions.

### 2.4. ALT and AST Activities

**Figure 5:**
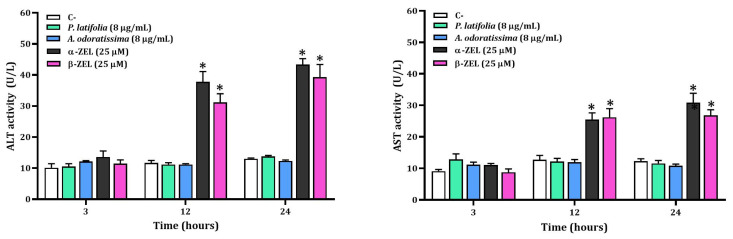
Effect of plant extracts, α-ZEL, and β-ZEL on ALT (**left**) and AST (**right**) levels measured in culture media of unexposed control cells and exposed cells. * Significant difference (*p* < 0.05) when compared to the negative control group (C-). α-ZEL and β-ZEL were used as positive controls.

The results of ALT and AST activities in HepG2 cells exposed to hydroalcoholic extracts from *P. latifolia* and *A. odoratissima* are presented in Figure 5. No statistically significant differences were observed in the mean ALT or AST activities induced by the extracts of the two plants when compared to the control cells. Moreover, it was found that both α-ZEL and β-ZEL exhibited high cytotoxicy. 

## 3. Discussion

Medicinal plants hold significant importance as therapeutic agents in folk medicine, especially in developing countries. In this study, semi-structured interviews and open discussions were conducted with six plant connoisseurs to gather detailed information on 100 plant species used in traditional medicine in San Basilio de Palenque, Colombia. From this extensive list, nine plant species were selected based on their wide usage in traditional medicine for various diseases. These nine species were then screened for cytotoxicity on HepG2 cells. Among the tested species, hydroethanolic extracts from the stems of *A. odoratissima* and *P. latifolia* were specifically evaluated for their potential anticancer activity using the normal liver epithelial cell line (HEKn) as a representative of normal human cells, to assess the toxicity of both extracts

Plants are known for their rich content of biologically active secondary metabolites, which play a positive role in human health by preventing and treating various diseases. Phenolic compounds, including flavonoids, are among the key compounds with beneficial properties such as antioxidant, anti-carcinogenic, cardioprotective, immune system support, anti-inflammatory, skin protective, and antibacterial activities [11]. Flavonoids, being the most abundant phenolics in plants, possess diverse bioactivities attributed to their potent antioxidant potential [11]. The results of this study revealed high levels of antioxidant compounds, specifically phenolics and flavonoids, in the hydroethanolic extracts. The highest concentrations of total phenols (>150 mg GAE/g DT) were found in *A. odoratissima*, *J. gossypiifolia* L., *P. peltatum* L., *D. incanum* (Sw.), and *D. unguis-cati* (L.) L.G. Lohmann. Regarding flavonoids, concentrations exceeding 30 mg RE/g DT were detected in *P. latifolia*, *D. incanum* (Sw.), and *A. odoratissima*. These findings align with a study conducted by Mariyammal et al. [12], which reported 3.2-fold lower phenolic content in the hydroalcoholic leaf extract of *Aristolochia tagala* compared to the species of the genus Aristolochia evaluated in this work (52.58 ± 0.06 mg GAE/g vs. 167.6 ± 1.3 mg GAE/g).

The screening of cytotoxic activity revealed that *A. odoratissima* and *P. latifolia* hydroethanolic extracts exhibited a high cytotoxic effect on HepG2 cells, with IC_50_ values of 54.2 µg/mL and 17.2 µg/mL, respectively, after 48 h of exposure. Previous studies have reported higher IC_50_ values (>200 µg/mL) for *A. odoratissima* methanolic extract on human kidney (HK-2) cells [13]. In San Basilio de Palenque, various connoisseurs have highlighted the usage of Aristolochia species in treating several diseases, including diabetes, cancer, inflammation, snakebites, viral and bacterial infections, and stomach problems, among others. These medicinal plants from the Aristolochia genus are not only utilized in America but also in Asian countries [12]. In Colombia, *A. odoratissima* is commonly known as ‘capitana’ [14]. In San Basilio de Palenque, it is primarily used for reducing inflammation-related pain and as an antidote against venomous animals, which aligns with the properties reported by Montiel-Ruiz et al. [15]. It was somewhat unexpected to find that the extract of *D. incanum* presented a very low IC_50_ (0.04 mg/mL) in relation to the total flavonoid content, which is within the same order of magnitude as the other extracts. However, this behavior could be due to several factors. First is the presence of one or several substances that can interfere with the DPPH assay, as has been documented for pigments [16], but this probably was not the case, as the extract had very little color. Second, substances different from flavonoids, such as alkaloids bearing free phenolic groups, can also exert antiradical activities [17].

*Picramnia latifolia* Tul. (Picramniaceae) is a plant species found in tropical regions. Extracts derived from this plant have been reported to possess anti-plasmodial effects [18]. Moreover, the *P. latifolia* extract exhibited a high selectivity index (>2), which could be attributed to its content of flavonoids and phenols. Interestingly, co-treatment with the *P. latifolia* extract and α-ZEL demonstrated a cytoprotective effect on HepG2 cells. It is worth noting that the evaluated extracts did not induce significant changes in the activity of liver damage marker enzymes (ALT and AST) when compared to the control group (untreated cells), suggesting the potential safety of both extracts for phytotherapeutic treatments.

The current uses and reported secondary metabolites for studied plants are presented in Table 3.

As demonstrated in this study, the investigated plants not only serve as valuable tools for managing symptoms of various diseases within the community but also hold potential for the development of bioeconomy-related ventures. Several plants have been identified as promising sources for the production of standardized extracts or as raw materials to extract high-value active compounds, such as luteolin, apigenin, quercitrin, quercetin, kaurenoic acid, hinokinin, and many others [15,31].

## 4. Materials and Methods

### 4.1. Plant Material

The study began with semistructured interviews and open discussions involving six plant connoisseurs who provided detailed information on 100 plant species commonly used in traditional medicine. From this extensive list, nine species were selected based on their widespread use in treating various diseases. Plant samples, including roots, stems, bark, or leaves, were collected from different areas in San Basilio de Palenque between August and September 2022. These plant specimens were then identified and deposited at the Colombian National Herbarium, with voucher specimens available (refer to Table 4). Additionally, the detailed information obtained from the interviews and discussions is provided in Table S1.

### 4.2. Extraction

The plant materials were air dried in the laboratory at room temperature (22–24 °C) and subsequently cut and powdered at the Ecotoxicology Laboratory, University of Cartagena (Cartagena, Colombia). The dried plant material was ground, and 200 g of the powdered material was subjected to extraction for 48 h using a mixture of ethanol (99.9%) and MilliQ water in a 70:30 ratio (*v*/*v*). After 24 h, the extract was filtered using Whatman paper, and the plant material was then subjected to another 24 h of hydroalcoholic extraction. The combined solutions were rotary evaporated to a volume of 40 mL and subsequently freeze-dried. Finally, the extracts were stored at −20 °C until further use [33].

### 4.3. Total Phenol Content

The total phenolic content was assayed according to Sánchez-Gutiérrez et al. [34] with modifications. Briefly, an aliquot of diluted hydroethanolic extract (0.25 mL) was added to 0.25 mL of distilled water and 0.125 mL of Folin–Ciocalteu reagent. The mixture was shaken and allowed to stand for 5 min before the addition of 0.375 mL of 20% (*w*/*v*) Na_2_CO_3_. After incubation in the dark for 2 h, absorbance at 760 nm was read versus a prepared blank using a Varioskan™ LUX Multimode Microplate Reader (Thermo Fisher Scientific, Inc., Waltham, MA, USA). The total phenol content of the hydroethanolic extract was expressed as milligrams of gallic acid equivalents (GAE) per gram of dry tissue (DT) (mg GAE/g DT) from a calibration curve with gallic acid. All samples were analyzed in three replicates.

### 4.4. Total Flavonoid Content

The measurement of total flavonoids was performed using the AlCl_3_ method, following the procedure described by Sánchez-Gutiérrez et al. [34] with slight modifications. In brief, a diluted hydroethanolic extract (0.4 mL) or a standard solution of rutin was mixed with AlCl_3_·6H_2_O solution (2%, 0.4 mL) and allowed to react for 3 min. The absorbance of the resulting mixture was measured at 415 nm against a blank using a Varioskan™ LUX Multimode Microplate Reader (Thermo Fisher Scientific, Inc., Waltham, MA, USA). The total flavonoid content was calculated as milligrams of rutin equivalents per gram of dry tissue (mgRE/g DT) using a calibration curve constructed with rutin. All samples were analyzed in triplicate.

### 4.5. Total Condensed Tannins

The evaluation of condensed tannins was conducted using the vanillin-H_2_SO_4_ method, following the methodology described by Sánchez-Gutiérrez et al. [34]. Briefly, diluted hydroethanolic extract (0.25 mL) was mixed with 1% (*w/v*) vanillin solution in methanol (0.25 mL) and 25% (*v/v*) H_2_SO_4_ (0.25 mL). The mixture was allowed to stand for 15 min, and the absorbance was measured at 500 nm against a methanol blank using a Varioskan™ LUX Multimode Microplate Reader (Thermo Fisher Scientific, Inc., Waltham, MA, USA). The total condensed tannin content was expressed as milligrams of (+) catechin equivalents per gram of dry tissue (mg CE/g DT). All samples were analyzed in triplicate.

### 4.6. DPPH• Free Radical Scavenging Assay

The free radical scavenging activity was determined using a standard kit (Bioquochem, Llanera, Asturias, Spain) following previously described methods by Alvarez-Ortega et al. [35]. A 100,000 µg/mL solution of the hydroethanolic extract in Milli-Q water was prepared. To assess the scavenging activity, 200 µL of DPPH• reagent was mixed with 20 µL of the extract (with gradually increased concentrations ranging from 0.125 to 6 mg/mL) and incubated at room temperature for 1 h. After incubation, the absorbance was measured at 517 nm using a Varioskan™ LUX Multimode Microplate Reader (Thermo Fisher Scientific, Inc., Waltham, MA, USA). A TroloxR standard curve was utilized to determine the TEAC (Trolox Equivalent Antioxidant Capacity) of the tested extract concentrations. The concentration of the extract solution that caused 50% inhibition of the DPPH radical (IC_50_) was determined from the nonlinear regression plot of the inhibition percentage against the concentrations.

### 4.7. Cell Line

The human hepatoma HepG2 cell line (ATCC-HB-8065) was cultured in EMEN medium (Quality Biological, Gaithersburg, MD, USA) supplemented with 10% FBS (Biowest, Riverside, MO, USA) and 1% penicillin/streptomycin (Sigma-Aldrich, St. Louis, MI, USA). The cells were maintained in a humidified atmosphere with 5% CO_2_ at 37 °C [36]. Primary HEKn cells were cultured in KM culture medium (ScienCell Research Laboratories, Carlsbad, CA, USA) supplemented with KGS 100× (ScienCell Research Laboratories, Carlsbad, CA, USA) and 1% penicillin/streptomycin solution (ScienCell Research Laboratories, Carlsbad, CA, USA). All experiments were conducted using cells in the logarithmic growth phase.

### 4.8. MTT Assay

The cell lines were seeded in 96-well plates at a density of 2 × 10^4^ cells per well and allowed to adhere for 24 h. The HepG2 cells were then treated with the extract at concentrations ranging from 500 to 3.9 µg/mL (1:2 dilutions) for 24 h. Subsequently, MTT solution was added to each well at a concentration of 3 mg/mL, and the plates were further incubated at 37 °C for 4 h. After incubation, 0.2 mL of DMSO was added to dissolve the formazan crystals, and the absorbance was measured at 620 nm using a spectrophotometric microplate reader (Varioskan™ LUX, Thermo Fisher Scientific, Inc., Waltham, MA, USA). Cell viability was determined by comparing the absorbance of treated cells to that of untreated cells. Three independent experiments were performed with four replicates each. Furthermore, the cytotoxicity of *P. latifolia* and *A. odoratissima* extracts on primary HEKn cells was assessed. The selectivity index (SI) was obtained calculated as the ratio of the IC_50_ value of noncancer cells to the IC_50_ value of cancer cells.

### 4.9. Cytoprotective Effects of P. latifolia and A. odoratissima Extracts against ZEN Metabolites

To assess the cytoprotective effect of *P. latifolia* and *A. odoratissima* extracts against ZEN metabolite-induced toxicity, a concentration of 8 µg/mL was selected for both extracts, which showed no significant cytotoxicity. The experiments consisted of treating the cells with a fixed concentration of *P. latifolia* extract (8 µg/mL) in combination with various concentrations of α-ZEL or β-ZEL (ranging from 0.4 to 100 µM, with 1:2 dilutions). The plates were then incubated for 24 h and 48 h at 37 °C in a 5% CO_2_ atmosphere, and cell viability was assessed using the MTT assay. Three independent experiments were conducted, with four replicates for each condition.

### 4.10. Biochemical Analysis

The activities of aspartate aminotransferase (AST) and alanine aminotransferase (ALT) were measured according to established methods [37,38]. The absorbance at 535 nm was measured using a Bio-systems BTS-350 spectrophotometer (Barcelona, Spain). The concentrations of AST and ALT were expressed as units per liter (U/L).

### 4.11. Statistical Analysis

The results are presented as the mean ± standard error of the mean (X ± SEM). The normality of the data was assessed using the Shapiro-Wilk test. Multiple data comparisons were performed using ANOVA, followed by Dunnett’s test. The IC_50_ values for each treatment were determined using nonlinear sigmoid curve fitting. Statistical significance was defined as *p* < 0.05. All statistical analyses were conducted using GraphPad Prism 8.0 software.

## 5. Conclusions

The biota of San Basilio de Palenque presents a promising source of anti-inflammatory plant species rich in flavonoids, phenolics, and tannins. *P. latifolia* shows potential for isolating natural products with anticancer properties, while the extract of A. odoratissima holds promise as an immunomodulator plant and a potential candidate for further investigations in developing natural compounds with immunomodulatory and anticancer properties. However, further studies are required to fully explore and understand the potential benefits of these plants.

## Figures and Tables

**Table 1:** Phenolic, flavonoid, and tannin contents, and DPPH free radical scavenging activity of hydroethanolic extracts from nine wild plant species collected in San Basilio de Palenque (Colombia).

Hydroethanolic Extract	Total Phenolic Content mg GAE/g DT	Total Flavonoid Content mg RE/g DT	Tannin Content mg CE/g DT	DPPH IC_50_ (mg/mL)
*P. latifolia*	145.5 ± 1.7	95.1 ± 0.5	73.5 ± 0.6	0.9
*J. gossypiifolia* L.	156.7 ± 1.4	17.7 ± 0.1	37.7 ± 0.7	1.2
*M. alceitoflia* Jacq	109.4 ± 0.6	7.1 ± 0.1	25.5 ± 0.5	2.2
*V. turbacensis* Kunth	96.7 ± 1.0	19.9 ± 0.1	30.5 ± 0.3	2.5
*P. peltatum* L.	157.9 ± 2.3	6.9 ± 0.5	32.8 ± 0.4	5.4
*R. communis* L.	131.5± 1.0	14.6 ± 0.8	19.3 ± 0.5	5.8
*D. incanum* (Sw.)	162.7 ± 4.9	65.1 ± 1.0	28.7 ± 0.5	0.04
*D. unguis-cati* (L.) L.G. Lohmann	151.3 ± 2.0	21.9 ± 0.4	22.0 ± 0.7	0.9
*A. odoratissima*	167.6 ± 1.3	31.4 ± 0.5	23.5 ± 0.8	2.6

**Table 2:** Selectivity index (SI) of hydroethanolic extracts on HEKn relative to HepG2 cell lines.

Hydroethanolic Extract	Exposure Time (h)	IC_50,_ µg/mL (95% CI)		SI
		HepG2	HEKn	
*P. latifolia*	24	128.9 (110.3–135.3)	160.0 (142.9–187.2)	0.4
	48	17.2 (12.5–19.9)	38.9 (31.5–40.5)	2.3
*A. odoratissima*	24	95.7 (110.3–135.3)	133.8(105.9–166.9)	1.4
	48	54.2 (48.9–60.2)	111.9 (88–164.5)	2.1

**Table 3:** Medicinal applications of plants and their reported chemical constituents.

Species	Current Uses	Reported Secondary Metabolites	References
*J. gossypiifolia*	Anti-inflammatory, anti-hemorrhagic, edematogenic, and antimicrobial. Latex used to treat wounds and bites of snakes.	Jatrophone, jatrophenone, jatrophatrione, Jatrophenone, curcusone A, japogadrol, multifolone.	[19,20]
*P. peltatum*	Anti-inflammatory, antimalarial, antiplasmodial, and antioxidant.	Nerolidylcatechol.	[21,22]
*M. alceifolia*	Anti-inflammatory, antileishmanial.	Episwertenol, α-amiryn, methyl commate.	[23,24]
*V. turbacensis*	Anticryptococcal.	Alpha-pinene (Bark oil); germacrene-D, delta-elemene (leaf oil), bornyl hydroxycinnamic esters (bornyl caffeate and bornyl ferulate).	[25,26]
*R. communis*	Anti-inflammatory properties. It is also used to treat liver infections, stomach ache, flatulence, constipation, colic, enteritis, fever, and headache, among others.	Kaempferol-3-O-β-D-xylopyranoside, kaempferol-3-O-β-D-glucopyranoside, kaempferol-3-O-β-rutinoside, quercetin-3-O-rutinoside, quercetin-3-O-β-D-xylopyranoside, quercetin, and rutin, among others.	[27,28]
*D. incanum*	Diuretic and anti-inflammatory.	6-C-galactosyl-8-C-glucosylapigenin, vicenin-2, 6-C-galactosyl-8-C-glucosylapigenin, 6-C-galactosyl-8-C-arabinosylapigenin, isoschaftoside, 6-C-arabinosyl-8-C-galactosylapigenin.	[29,30]
*D. unguis-cati*	Antipyretic, anti-inflammatory, and anti-tumoral. Potential hypocholesterolemic activity.	Chlorogenic acid, caffeic acid, ferulic acid, vanillinic acid, *p*-coumaric acid, rosmarinic acid, trans-cinnamic acid, luteolin, apigenin, quercitrin and quercetin.	[31]
*A. odoratissima*	Analgesic, antidote against snake venom.	Kaurenoic acid, hinokinin.	[15]
*P. latifolia*	Anti-malarial activity.	Picramniosides G and H, mayosides D and E, 6,8-dihydroxy-10-methyl-7H-benz[de]anthracen-7-one, 6,8-dihydroxy-4-methyl-7H-benz[de]anthracen-7-one, nataloe-emodin, chrysophanein, chrysophanol, 1,5-dihydroxy-7-methoxy-3-methylanthraquinone, pulmatin, 7-hydroxycoumarin, 7-hydroxy-6-methoxycoumarin, β-sitosterol.	[18,32]

**Table 4:** Information on plants used in this study collected in San Basilio de Palenque (Colombia).

Code	Plant Name	Family Name	Plant Part Used	Voucher Number
001	*Aristolochia odoratissima* L.	Aristolochiaceae	Stem	617813
003	*Jatropha gossypiifolia* L.	Euphorbiaceae	Stem	617821
004	*Piper peltatum* L.	Piperaceae	Stem	617811
005	*Malachra alceifolia* Jacq.	Malvaceae	Stem	617817
006	*Picramnia latifolia* Tul.	Picramniaceae	Stem	617812
007	*Verbesina turbacensis* Kunth	Asteraceae	Stem	617818
008	*Ricinus communis* L.	Euphorbiaceae	Stem	617814
010	*Desmodium incanum* (Sw.) DC.	Fabaceae	Stem, leaves	617819
012	*Dolichandra unguis-cati* (L.) L.G. Lohmann	Bignoniaceae	Stem	617820

## Data Availability

All data are included in the main text.

## Supplementary Materials

The following supporting information can be downloaded at: https://www.mdpi.com/article/10.3390/plants12142686/s1, Table S1: Code, common name, scientific name, medicinal use, part of the plant used of medicinal plants used by the knowers of San Basilio de Palenque, Bolívar, Colombia.
