# Supplementary material for: Cytotoxicity of Nine Medicinal Plants from San Basilio de Palenque (Colombia) on HepG2 Cells
Source: Plants (Basel). 2023 Jul 19;12(14):2686. doi: 10.3390/plants12142686 (PMC10383961; doi:10.3390/plants12142686)
Supplement: Supplementary file 1 [file plants-12-02686-s001.zip › plants-2476978-supplementary.pdf]

Table S1. Code, common name, scientific name, medicinal use, part of the plant used of medicinal plants used by the knowers of San Basilio de Palenque, Bolívar, Colombia.

Code	Common name	Scientific name	Family	Medicinal use	Plant part used
**001**	Capitana	*Aristolochia odoratissima* L.	Aristolochiaceae	Anti-inflammatory and animals bite remedy	Stem
**002**	Salvia	*Croton* sp.	Euphorbiaceae	Anti-inflammatory and flu	Stem and leaves
**003**	Tua tua	*Jatropha gossypiifolia* L.	Euphorbiaceae	Anti-inflamatorio, cholesterol, diabetes and fungus	Stem and leaves
**004**	Santa María	*Piper peltatum* L.	Piperaceae	Anti-inflammatory	Stem and flower
**005**	Malva	*Malachra alceifolia* Jacq.	Malvaceae	Anti-inflammatory and cystitis	Stem and leaves
**006**	Quina	*Picramnia latifolia* Tul.	Picramniaceae	Anti-inflammatory, spasms, pain, and fever	Stem
**007**	Selvatana	*Verbesina turbacensis* Kunth	Asteraceae	Anti-inflammatory	Stem
**008**	Higuereta	*Ricinus communis* L.	Euphorbiaceae	Anti-inflammatory	Stem and leaves
**009**	Cincollagas	*Passiflora* sp.	Passifloraceae	Anti-inflammatory, fever and flu	Stem
**010**	Calilla de perro largo	*Desmodium incanum* (Sw.) DC.	Fabaceae	Anti-inflammatory	Stem and leaves
**011**	Contra gavilana	*Schultesia lisianthoides* (Griseb.) Benth. & Hook. f. ex Hemsl.	Gentianaceae	Anti-inflammatory and parasites	root, stem and leaves
**012**	Uñita gato	*Dolichandra unguis-cati* (L.) L.G. Lohmann	Bignoniaceae	Anti-inflammatory	--
**013**	Caga de pajarito	*Phoradendron* cf. *quadrangulare* (Kunth) Griseb.	Santalaceae	Anti-inflammatory, diabetes and poor circulation.	Stem
**014**	Albaca silvestre	*Hyptis suaveolens* (L.) Poit.	Lamiaceae	Anti-inflammatory	Stem and leaves
**015**	Caña de mico	*Costus* cf. *Spiralis* (jacq.) Roscoe	Costaceae	Anti-inflammatory and kidney diseases	Stem
**016**	Malambo	*Croton malambo* H.Karst.	Euphorbiaceae	Snake bite and pain	leaves and cortex

**017**	Lengua suegra	*Kalanchoe poincarei* Raym.-Hamet & H.Perrier	Crassulaceae	Pains	leaves
**018**	Colicenci	*Cordia* sp.	Boraginaceae	Flu, headache and suffocation	leaves
**019**	Cruzeta	*Quassia* sp.	Simaroubaceae	Flu, fever and body aches	stem, cortex and leaves
**020**	Malamadre	*Kalanchoe* sp.	Crassulaceae	Insect bites and snake bites	Stem
**021**	Coquito	*Cyperus luzulae* (L.) Retz.	Cyperaceae	Pain and hemorrhoids	Stem and leaves
**022**	Santa María	*Baccharis trinervis* Pers.	Asteraceae	blows and pain	Stem and leaves
**023**	Totumo	*Crescentia cujete* L.	Bignoniaceae	flu and phlegm (mucus)	Fruit
**024**	Matarraton	Gliricidia sepium (Jacq) Kunth ex Walp.	Fabaceae	Fever, allergies in the body and pains	leaves
**025**	Anamú	*Petiveria alliacea* L.	Phytolaccaceae	Fever	leaves and stems
**026**	Pringamoza blanca	*Cnidoscolus urens* (L.)	Euphorbiaceae	Mucus, stones, and cysts in the vagina	Root
**027**	Bejuco cadena	*Bauhinia glabra* Jacq.	Fabaceae	Phlegm, mucus and blood pressure	Root
**028**	Escobilla menuda	*Scoparia dulcis* L.	Plantaginaceae	Diarrhea, vomiting, toothache and parasites	Root, Stem and leaves
**029**	heliotropo indio, cola de alacrán	*Heliotropium indicum* L.	Boraginaceae	Vomiting, tummy ache, parasites and phlegm	Root, Stem and leaves
**030**	Bicho, palo zorrillo	*Senna obtusifolia* (L.) H.S. Irwin & Barneby	Fabaceae	Vomiting and diarrhea	Root
**031**	Moringa	*Moringa oleifera* Lam.	Moringaceae	phlegm (mucus)	Fruit, leaves and seed
**032**	Chocolatillo	*Chomelia spinosa* Jacq.	Rubiaceae	Flu and fever	Stem and leaves
**033**	Ariza	*Brownea ariza* Benth.	Fabaceae	Hemorrhage	Cortex and flower
**034**	Julio	*Ichthyothere* sp.	Asteraceae	wounds and bleeding	leaves
**035**	Guarumo	*Cecropia peltata* L.	Urticaceae	Prostate pain and spasm	leaves
**036**	La leona, lengua de culebra	*Barleria lupulina* Lindl.	Acanthaceae	muscle aches	leaves
**037**	Pata de vaca	*Bauhinia pauletia* Pers.	Fabaceae	Pain, calculus and prostate	Stem

**038**	Papaya	*Carica papaya* L.	Caricaceae	Sinusitis	Flower and fruit
**039**	Contrayerba	*Dorstenia contrajerva* L.	Moraceae	Insect bite	Root and flower
**040**	Jagua	*Senna reticulata* (Willd.) H.S.Irwin & Barneby	Fabaceae	Purgative	leaves
**041**	Toronjil, albahaca lima	Ocimum americanum L.	Lamiaceae	Flu	Stem and leaves
**042**	Matandrea	*Renealmia* sp.	Zingiberaceae	Fever	leaves
**043**	Coralillo, lágrimas de Cristo	*Russelia equisetiformis* Schltdl. & Cham.	Scrophulariaceae	Pains	Stem and leaves
**044**	Nim, Neem, Paraiso de la India	*Azadirachta indica* A.Juss.	Meliaceae	Control sugar levels	Stem and leaves
**045**	Mango de Puerco	*Mangifera indica* L.	Anacardiaceae	Pains	leaves
**046**	Orégano	*Origanum vulgare* L.	Lamiaceae	Ear pain and flu	leaves
**047**	Pítamo real	*Euphorbia tithymaloides* L.	Euphorbiaceae	toothache and earache	leaves
**048**	Guanábana	*Annona muricata* L.	Annonaceae	control cancer	leaves
**049**	Manito de Dios	*Kalanchoe pinnata* (Lam.) Pers.	Crassulaceae	Pain and insect bites	leaves
**050**	Uvita	*Cordia dentata* Poir.	Boraginaceae	Allergy, conjunctivitis and toothache	Flower and fruit
**051**	Balsamina	*Momordica charantia* L.	Cucurbitaceae	allergies and diabetes	leaves
**052**	Bejuco alambrito	*Funastrum* sp.	Apocynaceae	Allergy and snake bite	Stem and leaves
**053**	verdolaga, Joyweed	*Alternanthera* sp.	Amaranthaceae	Gastritis	Stem and leaves
**054**	Cierrateputa	*Mimosa pudica* L.	Fabaceae	Snake bite, poor blood circulation and diabetes	Root and stem
**055**	Menta	*Mentha x piperita* L.	Lamiaceae	gastritis and phlegm	Stem and leaves
**056**	Singa mochila	*Justicia secunda* Vahl	Acanthaceae	kidneys and cholesterol	leaves
**057**	Cadillo de perro bolsita	*Priva lappulacea* (L.) Pers.	Verbenaceae	Pain, cirrhosis and open wounds	Stem and leaves

**058**	Azahar de la india	*Murraya paniculata* (L.) Jack	Rutaceae	Toothache and fever	leaves, flower and fruit
**059**	Acetaminofen, boldo	*Plectranthus neochilus Schltr*	Lamiaceae	Headache, fever, gastritis and blood pressure	Root, stem y leaves
**060**	Culantro	*Eryngium foetidum* L.	Apiaceae	stomach constipation	Root, stem y leaves
**061**	Guandú	*Cajanus cajan* (L.) Huth	Fabaceae	fever and mucus	Root, stem y leaves
**062**	Matimba	*Annona purpurea* Moc. & Sessé ex Dunal	Annonaceae	Fever and headache	leaves and fruit
**063**	Flor de muerto	*Caesalpinia pulcherrima* (L.) Sw.	Fabaceae	Phlegm and chronic flu	Flower
**064**	Aguacate	*Persea americana* Mill.	Lauraceae	Fertility	Stem, leaves and fruit
**065**	Campano	*Samanea saman* (Jacq.) Merr.	Fabaceae	body aches and flu	Cortex
**066**	Olivo	*Quadrella odoratissima* (Jacq.) Hutch.	Capparaceae	fever and flu	Stem and leaves
**067**	Ahuyama	*Cucurbita maxima* Duchesne	Cucurbitaceae	Body pain	Stem and leaves
**068**	Toporopo	*Physalis angulata* L.	Solanaceae	Gastritis	Root, Stem and leaves
**069**	Jobo de cerca	*Spondias mombin* L.	Anacardiaceae	eye discomfort	exudate
**070**	Guamacho	*Pereskia guamacho* F.A.C.Weber	Cactaceae	chronic flu	Stem and leaves
**071**	Flor de Jamaica	*Hibiscus sabdariffa* L.	Malvaceae	Headache	Root and stem
**072**	Almendra	*Terminalia catappa* L.	Combretaceae	Muscle pain	stem and leaves
**073**	Cañandonga	*Cassia grandis* L.f.	Fabaceae	Anemia and platelets	Fruit
**074**	Sábila	*Aloe vera* (L.) Burm.f.	Xanthorrhoeaceae	Blows and flu	leaves
**075**	Bleo de puerco	*Amaranthus spinosus* L.	Amaranthaceae	Fever	root, stem and leaves
**076**	Cola babilla	*Epiphyllum* sp.	Cactaceae	expel mucus	stem
**077**	Hierbamora	*Solanum americanum* Mill	Solanaceae	allergies	root, stem and leaves
**078**	Raíz de murciélago	*Chiococca* sp.	Rubiaceae	allergies	Tuber
**079**	Ceiba blanca	*Hura crepitans* L.	Euphorbiaceae	wounds	Seed and exudates

**080**	Milagrosa	*Verbesina pterophora* S.F.Blake	Asteraceae	poor blood circulation	stem and leaves
**081**	Caracolí	*Anacardium excelsum* (Bertero ex Kunth) Skeels)	Anacardiaceae	Anemia and fertility	Fruit
**082**	Caraña	*Bursera graveolens* (Kunth) Triana & Planch.	Burseraceae	Fever, flu and headache	bark and leaves
**083**	Cedro	*Cedrela odorata* L.	Meliaceae	cough and body pain	leaves
**084**	Achiote	*Bixa orellana* L.	Bixaceae	pain and burns	Leaves and fruit
**085**	Mora blanca	*Maclura tinctoria* (L.) D. Don ex G. Don	Moraceae	Parasites	bark and exudates
**086**	Gusanero	*Astronium graveolens* Jacq	Anacardiaceae	fever and flu	leaves
**087**	Dividivi	*Caesalpinia coriaria* (Jacq.) Willd.	Fabaceae	Wounds and sores on the skin	seed
**088**	Plátano	*Musa* sp.	Musaceae	ulcer	stem
**089**	Maíz	*Zea mays* L.	Poaceae	kidneys and colon	flower
**090**	Roble	*Tabebuia rosea* (Bertol.) Bertero ex A.DC.	Bignoniaceae	Headache, sinusitis and fever	leaves
**091**	Bejuco batea	*Dolichandra quadrivalvis* (Jacq.) L.G.Lohmann	Bignoniaceae	Sinusitis	leaves and seed
**092**	llantén	*Plantago major* L.	Plantaginaceae	pains	leaves
**093**	Cotorrera	*Ocimum campechianum* Mill.	Lamiaceae	Fever, blurred vision and allergy	Flower
**094**	Hombre solo	*Acacia collinsii* Saff.	Fabaceae	pain and allergy	Stem and leaves
**095**	Cajón	*Hibiscus rosa-sinensis* L.	Malvaceae	poor blood circulation	leaves, stem
**096**	Algarrobo	*Hymenaea courbaril* L.	Fabaceae	Hemoglobin	Frut
**097**	Cigarrón	*Senna fruticosa* (Mill.) H.S.Irwin & Barneby.	Fabaceae	Headache	Stem and leaves

**098**	Katriana	*Hyptis capitata* Jacq.	Lamiaceae	skin spots	Root, stem, and leaves
**099**	Disulina	*Chamaecostus* sp.	Costaceae	Diabetes and pain	leaves
**100**	Sangrina	*Curcuma longa* L.	Zingiberaceae	bleeding and pain	leaves and tuber
